# “I did whatever they wanted me to do”: a qualitative secondary analysis using reproductive justice to explore sexual violence among justice-involved Latina mothers

**DOI:** 10.1186/s12889-022-13865-8

**Published:** 2022-07-30

**Authors:** A. D. Crawford, K. McGlothen-Bell, L. M. Cleveland

**Affiliations:** 1 The University of Texas at Austin, School of Nursing, 1710 Red River Street, Austin, TX 78712 USA; 2 The University of Texas Health at San Antonio School of Nursing, 7703 Floyd Curl Drive, Mail Code 7951, San Antonio, TX 78229-3900 USA

**Keywords:** Latinas, Mothers, Sex-work, Sexual violence, Criminal justice, Reproductive justice

## Abstract

**Background:**

One in three women experience sexual violence during their lifetime; however, little is known about this phenomenon with respect to justice-involved Latina mothers. Using the reproductive justice framework as a theoretical lens, we examined sexual violence in Latina mothers who had experienced incarceration and were thus involved in the justice system.

**Methods:**

This was a secondary analysis of a qualitative data set. The reproductive justice framework provided a theoretical lens for examining the women’s rights to bodily autonomy, to have or not have children, and to live in safe, sustainable environments given the intersection of incarceration and sexual violence.

**Results:**

Women (*N* = 12) recounted their experiences of sexual violence after having been incarcerated. Incarceration and resulting sexual violence led to discrimination, limited bodily autonomy, sexual exploitation, substance use, depression, anxiety, re-traumatization, recidivism, underreporting of violence, underutilization of healthcare resources, strained relationships, family separation, and unsafe environments.

**Conclusions:**

More research is needed to understand the social, economic, and political contexts that perpetuate sexual violence among justice-involved women. Universal healthcare, participatory research, changing cultural mindsets, decriminalization of sex work, and more comprehensive tracking and prosecution of sexual predators may be key to ending sexual violence in justice-involved mothers.

## Introduction

Globally, one in three women experience sexual violence during their lifetime [1], even though sexual violence is preventable. Sexual violence is defined as an abhorrent “sexual act, attempt to obtain a sexual act, or other act directed against a person’s sexuality using coercion, by any person regardless of their relationship to the victim, in any setting [2]. This includes rape, defined as the physically forced or otherwise coerced penetration of the vulva or anus with a penis, other body part or object, attempted rape, unwanted sexual touching and other non-contact” [2]; it also includes coercion or exploitation of sexual acts within underrepresented groups, among whom are individuals who engage in sex work [3, 4]. The need to address sexual violence is well-documented; it is both a persistent human rights violation and a major public health concern. In the U.S., an average of 463,634 individuals experience an act of sexual violence every year, or one person every 68 seconds [2, 5].

Sexual violence comes with substantial costs that affect survivors’ ability to work, prosper, interact with their communities, or care for their children [2]. Survivors of sexual violence have higher rates of morbidity and mortality, depression, anxiety, posttraumatic stress disorder, substance use, chronic stress, preterm birth, children with low birthweight, hypertension, suicide, and homicide [2]. Those most at risk for sexual violence identify as women of color and have histories of family violence, childhood trauma, low educational level, pay inequality, limited access to healthcare, and food and housing insecurity [2]. Individuals with disabilities, and those who live in areas with discriminatory laws or in regions of the world that reinforce negative masculine behavior, such as cultures that victim blame during instances of reported sexual misconduct, have limited laws that protect women’s equality, or that place pressure on men to be controlling over women, are also at increased risk [2]. A context of sexual violence also affects children’s health and well-being both emotionally and behaviorally, even in later life [2].

In a recent review, Karlsson and Zielinski [6] have suggested that lifetime exposure to sexual violence contributes to incarceration, along with substance use disorder and re-traumatization. Women with a history of arrest have a higher prevalence of childhood and lifetime sexual violence than do women in the general population, and they have unique emotional and psychosocial needs; the lack of policies and programs to address those needs may contribute to lifelong and intergenerational trauma, mood disorders (post-traumatic stress disorder, depression, and anxiety), economic barriers, and gender-specific circumstances related to substance use disorder and arrest. Gender specific circumstances related to substance use disorder and arrest for women include histories of experiencing or witnessing violence, often in the form of sexual violence which serves as a precursor to their substance use disorders and eventual arrest [6]. Yet as Karlsson and Zielinski show, although sexual violence against women has been broadly studied, little research has focused on the role of incarceration in perpetuating sexual violence. Indeed following arrest, incidences of sexual violence committed against women by those holding positions of power and authority may be increased [6].

No other system in the U.S. stratifies individuals by class and race to the extent that the nation’s criminal justice system does [7]. Over the past 40 years, the incarceration of U.S. women of color who are of reproductive age (12–50 years) has increased by more than 800% [8, 9]. In South Central Texas, women of reproductive age who self-identify as Latina are the majority ethnic group (Hispanic or Latino(a) 64.7%, white no Hispanic origin 24%, Black or African American 6.8%, American Indian 0.7%, Asian 3%,) [10], yet they experience higher rates of socioeconomic disadvantage, morbidity, and mortality than do their white counterparts [8]. They also experience high rates of arrest (178 per 100,000), and 44% are rearrested within the first year after release from incarceration [8, 11]. In this study, we therefore provide a secondary analysis of data on the experiences of sexual violence among justice-involved Latina mothers in South Central Texas. In this analysis, the term incarceration implicates justice system-involvement, which includes the many ways in which women encounter the criminal justice system: (a) arrest, (b) detainment, (c) charges with and/or convictions of a crime, (d) required community supervision (probation), and (e) court-mandated completion of drug treatment programs. These types of justice-system involvement, along with reentry into society, are often met with significant challenges to reproductive and civil rights [12].

### The reproductive justice framework

**Fig. 1:**
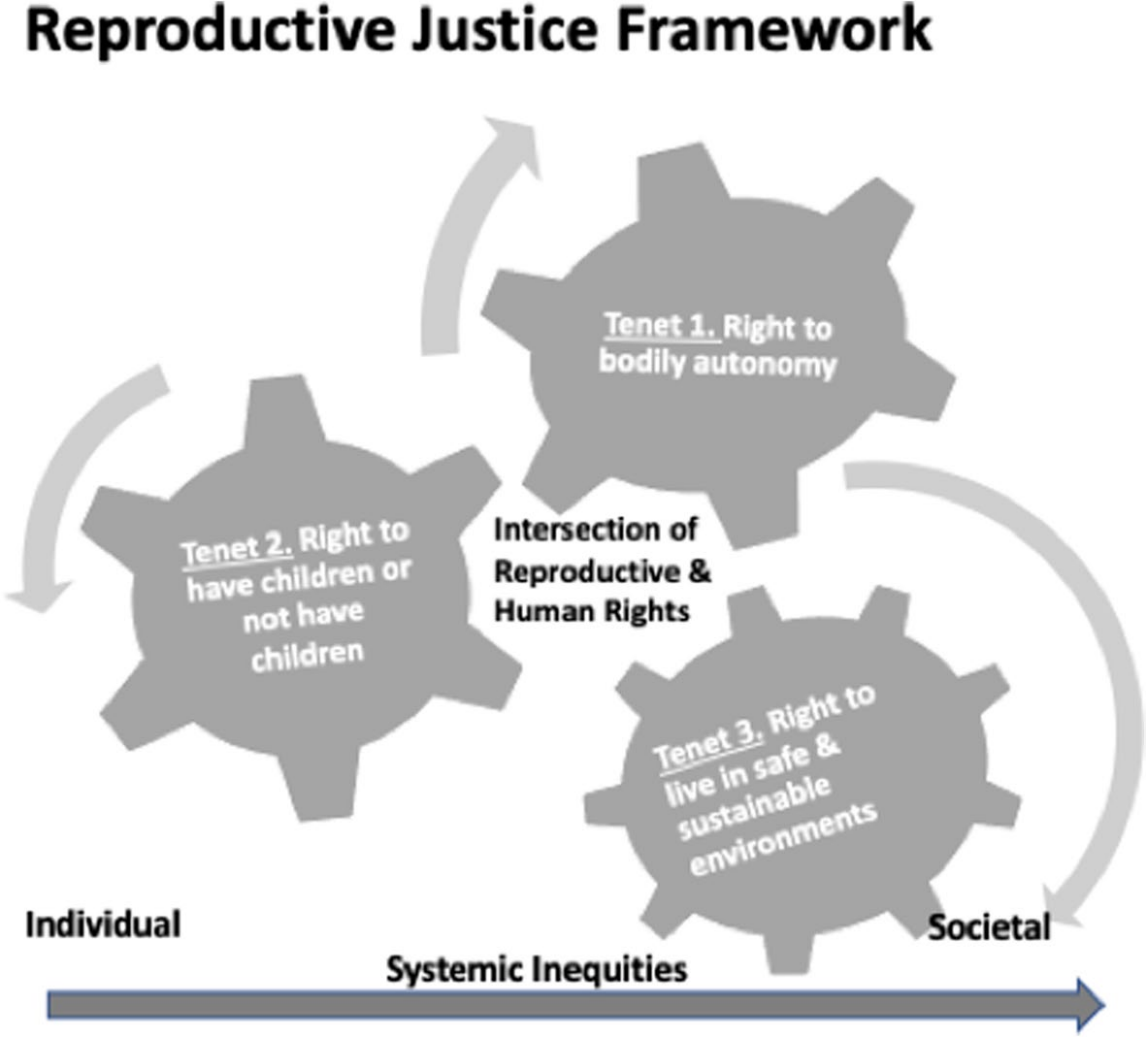
Reproductive justice

This study is informed by the framework of reproductive justice, a framework created by women of color and rooted in a broader framework for women’s human rights as well as in black feminist theory [9] (See Fig. 1). Reproductive justice allows the critical analysis of a population’s reproductive and civil rights. It has three primary tenets: (1) the right to bodily autonomy, (2) the right to have or not have children, and (3) the right to parent one’s children in safe, sustainable environments (Ross, 2017). At the same time, reproductive justice illuminates the experiences of those who have gone unheard, while allowing for a systematic analysis of the power and privilege that punitively regulate reproduction in underserved groups [7, 9]. Issues of gender, class, and race are central to reproductive justice [9], and the reproductive justice framework has been used to evaluate barriers and historical nuances of authority associated with the criminal justice system, law enforcement, housing, child welfare, and public assistance [7].

The framework of reproductive justice has transformed the field of reproductive rights by re-conceptualizing reproductive “choice” as “justice” for underserved groups [7, 9]. Prior research has conducted secondary analysis’ or written research commentary’s applying the reproductive justice framework to explore how women influenced by arrest have limitations to their reproductive rights and autonomy [13, 14]. Historically, justice has been left out of policy and program initiatives designed for populations of color [9], and so reproductive justice offers an appropriate framework for our investigation of sexual violence in justice-involved Latina mothers. The framework allows us to address gaps in the literature specific to the intersections of incarceration, sexual violence, gender, class, race, and reproductive and civil rights.

## Methods

We conducted a qualitative descriptive study in December of 2020 [12] to answer the following research question: What are the experiences of Latina mothers impacted by incarceration in the South-Central Texas region? Qualitative description was an ideal methodology for the original study, which was exploratory [15]. Our results revealed extensive data for sexual violence, which warranted the present secondary analysis to bring greater awareness to this violation of reproductive and civil rights and give voice to the participants who shared their personal accounts. This secondary analysis addressed the following research questions: (1) What are the sexual violence experiences of Latina mothers influenced by incarceration? (2) What influence do these experiences have on the women’s right to bodily autonomy, right to have or not have children, and right to parent their children in safe, sustainable environments?

### Sampling and inclusion criteria

The original study was approved by our university’s Institutional Review Board prior to data collection. A total of fourteen women were screened. One was excluded because they did not identify as Latina and another was not enrolled because they never followed up for the interview process. We recruited and enrolled a convenience sample (*N* = 12) of justice-involved women. No participants dropped out of the study. Participants were (a) at least 18 years of age; (b) self-identified as Latina; (c) pregnant or parenting children; (d) with a history of arrest; (e) with a judgment of guilty, a no contest plea, or deferred adjudication; (f) released from county jail, a detention center, state, or federal prison; and (g) currently or previously on adult probation, on parole, or in a court-mandated drug treatment program. Participants had to meet all the criteria with the exception of (g) to be included in the study. To recruit participants, we posted flyers with information about the study in (a) the county jail annex where individuals were released from county jail, (b) county adult probation offices, and (c) our local mental health authority, which houses the city’s largest methadone program. We also recruited through snowball sampling and social media (i.e. Facebook and Instagram). Interested participants contacted the principal investigator via telephone to be screened for eligibility, a process that took approximately 10 minutes. Steps were taken to address instances of emotional distress or re-traumatization such as providing the participants with telephone numbers to crisis hotlines and local counseling or crisis centers. In addition, in the event information was shared that seemed to threaten the participant’s well-being, measures were in place for the participant to be referred to a counselor or their case worker, however, there were no referrals needed at the conclusion of the interviews.

### Data collection

To maintain confidentiality, personal identifying information was not collected at enrollment. Instead, we read information sheets about the study to the participants and obtained their verbal consent which was approved by the University of Texas Health San Antonio ethical committee (protocol number: HSC20200738H). We then sent the information sheets to the participants via text message or email so that they would have a record of the study’s purpose and their consent. Due to the ongoing Covid-19 pandemic, we offered to meet with our participants either online via Zoom or by phone. All participants opted to use the phone, without any video. Prior to starting the telephone interviews, we asked the participants if they were in a space that was safe and private. Only one participant had to reschedule her interview to ensure she was in a private area to discuss her study. Another participant had to recontact the P.I. half-way through her interview to relocate to a private space.

We collected data in semi-structured, in-depth individual interviews, using an interview guide with open-ended questions; probe questions were added when needed, to ensure richness of data (see Table 1). We followed Sandelowski’s (2000) [15] recommendations when creating the interview guide to fit our qualitative descriptive methods. We started with broad questions asking the women what is was like being a mother following arrest to get as much data about their experiences using a ground-up approach. We then used probe questions for the women to expand on topics they mentioned that seemed to align with the reproductive justice framework. Sexual violence was not in our initial interview guide, however, that was an emerging theme within the data, therefore, we tailored the probe questions and asked others about their experience with sexual violence or coercion. We then member checked and recontacted one participant from earlier in the interview process to check with them if they had similar experiences of sexual violence and coercion.

**Table 1:** Participant interview guide

	Questions
**1**	Describe your experiences of being a mother following arrest?
**2**	What are the biggest challenges of parenting after arrest?
**3**	Describe a typical day in your life?
**4**	What are your barriers to healthcare?
**5**	What would make it easier for moms following arrest?
**6**	What is most important to you?
**7**	What worries you in regard to your children
**8**	What worries do you have regarding your health or the health of your family?
**9**	What about your health is most important to you?
**10**	Can you share with me what you want most for the future?
**11**	Is there something else you think I should know that I haven’t already asked?
**12**	Probe questions

Each interview lasted approximately 60–120 minutes, with one participant requiring a follow-up interview to further clarify her data. The interviews were audio recorded and transcribed verbatim by a professional transcription service. Each participant received a $20 gift card as compensation upon completion of her interview.

### Data analysis

We followed Sandelowksi’s (2000) [15] recommendation for thematic content analysis of the data. Thematic analysis began with reading the transcripts line by line to familiarize ourselves with the data.. The first level of coding consisted of codes for participants’ words and phrases specific to sexual violence, with key phrases that described instances of sexual violence entered in the margins of a Word document. For the second level of coding, using direct quotes from the participants, we developed themes from the coded text that matched the three tenets of reproductive justice: 1) the right to bodily autonomy, 2) the right to have or not have children, 3) the right to parent one chooses in safe and sustainable environments. The codes and themes were then reviewed and discussed by open discussion and refined by modifying which theme served as the exemplar by the three members of the research team, all of whom are experienced qualitative researchers. When disagreements occurred, discussion continued until a consensus was reached. There was no use of qualitative analysis software.

## Results

Three themes, presented in Table 2 with corresponding tenets of reproductive justice, emerged from the data: (1) They definitely take advantage, (2) You’re never going to get these kids, and (3) Why can’t I get out of this hell I’m living in?

**Table 2:** Themes

	Theme	Tenets of Reproductive Justice
**1**	*They definitely take advantage*	Tenet 1: The right to bodily autonomy
**2**	*You’re never going to get these kids*	Tenet 2: The right to have or not have children
**3**	*Why can’t I get out of this hell I’m living in?*	Tenet 3: The right to parent and live in safe and sustainable environments

The participants’ demographic data is presented in Table 3. All of the women (*N* = 12) had at least one felony charge and had been detained in county jail. Almost all (*n* = 11) had been on adult probation. Half of the women had attended only middle school and had a seventh (*n* = 2) or eighth (*n* = 4) grade education: 9 of the women (75%) reported an annual income of less than $9000 per year.

**Table 3:** Maternal demographics

Participants	N	Range	Mean
Number of Participants	12		
Age			
< 31	1		
32–38	5		
Older than 39	6		
Ethnicity			
Latina	12		
Relationship Status			
Single	7		
Living with Partner	4		
Married	1		
Number of Children			
< 4	6	1–4	2.5
Greater than 5	6	5–8	5.5
Highest Level of Education Completed			
Middle School	6		
High School	1		
Associates/ Certification	4		
Bachelors	1		
Employment Status			
Unemployed	10		
Part-time	0		
Full-time	2		
Annual Income			
Less than $9000	9		
$10,000–$19,000	2		
$20,000–$29,000	0		
$30,000–$39,000	1		
Number of Times Arrested			
		1–19	9
Nature of Arrests			
Low-level Drug	8		
Petty theft	7		
DUI	1		
Traffic Tickets	2		
Prostitution	2		
Assault	1		
County Jail	12	18 hrs. - 22mo.	11 mo.
State Prison	2	1 mo. – 2.2 yrs.	13 mo.
Federal Prison	0		
Community Supervision	11	2–5 yrs.	2.5 yrs.
Transition home	2	9–12 mo.	10.5 mo.
Misdemeanor	11		
Felony	12		

### Theme 1: they definitely take advantage

#### Broader community

The first theme, they definitely take advantage, reflected the right to bodily autonomy. The women described how the profound poverty and discrimination that resulted from their arrest, charge, and/or conviction limited their control over their own bodies. This, in turn, left them particularly vulnerable to sexual violence that resulted in feelings of “not [being] human.” One woman described how she was treated “inhumanely” following arrest: “It’s negative and bad. It’s bad in every aspect. I understand that we broke the law, but we’re still human.”

Another woman explained how she became a target of physical abuse once others discovered that she had been arrested for drug possession: “They would take me seriously until they heard I was a drug addict. The fact that I was on drugs meant that it was okay for someone to beat on me.” Following arrest, the women collectively described being in an even more vulnerable position than before, forced to “do whatever [people] wanted them to do”—another example of limited bodily autonomy.

#### Romantic partners

Often this vulnerability was exploited by individuals whom the women should have been able to trust. For example, one woman described the sexual violence she experienced at the hands of her boyfriend after she was released from jail:

He forced me down onto the bed on my stomach, ripped off my underwear, he was drunk, and he raped me. I was screaming out, “Help,” and he was covering my mouth and punching the back of my head, doing his thing. I thought I was gonna die. He pulled out a gun, held a gun to my head, finished, and then passed out.

After being influenced by arrest, women were often forced to go back to abusive partners because finding steady employment and housing is difficult. This woman described being held captive by her boyfriend while being physically and sexually assaulted for 10 days:

I was running from him throughout the house. He was like, “Come here. Let’s make this even better in blood.” He got a chair, sat me on the chair. He duct-taped my legs, my arms and my mouth about 10 times. He started shooting and hitting me with the gun. He really messed up my face. He threw me in the closet for two days. [The sexual violence] went on for about 10 days.

The women also described being forced into “escorting” for money by boyfriends on whom they relied for survival. Like so many in the sample, one woman described how these men, with whom she sought safety, had sexually exploited her for their own financial gain. As a result, many of the women turned to substance use as a means of coping with their trauma:

Escorting for them ... just selling my body to give them the money. One of the guys got me into strip clubs. I started dancing, and all the money I would make would go to them. That led to my addiction. So, money that I did make—half of it would go to them, and the other half would go to my drugs.

Although many of the women had been involved in sex work prior to arrest, they described the devastation of returning to that work upon release just to survive the economic hardship resulting from incarceration. One woman explained that being a sex worker made her feel “ugly”: “I just felt like I have to oversex myself. My self-esteem was low. I felt useless. I just felt ugly.” In many cases, the women’s partners took full advantage of their vulnerability by sexually exploiting them further through sex work, which became more “dangerous” and “violent” over time due to their inability to defend themselves. Their partners also used the women’s prior criminal records against them, knowing that they had limited social safety nets and resources. One woman recounted being held at gunpoint by a client while her boyfriend waited outside the hotel room:

[My boyfriend] took me on this website, on the internet. Whenever these guys would call me, they would take me over there to have sex. They [her boyfriend] would wait outside and collect the money. Then they’d just take me to the next one, and the next one, and it just got really ugly. It’s very scary. ‘Cause some situations [clients] would hold me at gunpoint.

Isolation was a common tactic that partners used to control the women. One woman described how, after her release from jail, her boyfriend moved her to Hawaii, away from her support network back in Texas, giving him even greater control over her body:

[My boyfriend] used [my vulnerability after arrest] to his advantage. He got a job in Hawaii. He was like, “I want you to come with me,” so of course, who wouldn’t wanna move to Hawaii? I took my son, and we went to Hawaii. [My boyfriend] immediately was like, “I made you a website. I made you an account. I already got you these clients.” It was really sick.

#### Men in positions of power

In addition to the sexual violence perpetuated by their partners, the women described being vulnerable to persons in positions of authority, such as those working in the jail system: guards, pretrial bond officers, police officers, and court appointed attorneys. The women explained that, while they were in custody, these individuals “used their power” to satisfy their own sexual desires. One woman described meeting with a pretrial bond officer who became sexually inappropriate once he discovered that she had been charged with prostitution, an offense attached to significant discrimination according to the women: “They literally just think that they can touch me—they can touch me without asking me or anything like that. I don’t know. It’s just weird how they think they can just do stuff like that to you.” Another woman recalled how her court-appointed attorney had coerced her into having sex with him by threatening her with jail time if she refused to comply. She described the first of many times he sexually assaulted her:

He started taking my clothes off. I was like, “Whoa, what are you doing? I just wanna leave.” He was like, “No.” He started having sex with me. I was just crying. I was telling him, “No. Stop, please.” He started just having sex with me. Then after that he was like, “You better not say nothing because I can make it a lot worse for you.”

A similar story was echoed by another woman, who described inappropriate sexual conduct of the guards in the county jail. She felt powerless to tell anyone:

I remember there was this one guard. She was trying to talk to me. When I would go and take a shower, she would [follow me]. I just felt like she would do that on purpose. She would go and look behind the shower wall. There’s not much that you could do or say ‘cause you’re in jail. Your word doesn’t really matter when you’re in jail. Who are they gonna believe? Are they gonna believe these convicts, or are they gonna believe somebody that’s in uniform?

Yet another woman shared this experience:

Whenever you’re passing through a different hallway or corridor, [the guards] have to pat you down and check to make sure you don’t have any type of contraband. They definitely take advantage of that and feel you up. I wanna say probably it happens more with females than it does with males.

Even women who might not have personally experienced this abuse of power knew of women who had. One said that her incarcerated female friend had disclosed that a guard was forcing her to have sex with him: “She said, ‘Look, that’s the guard that I have sex with.’ I said, ‘What do you mean?’ She said, ‘Yeah, he takes me somewhere [in the back] to have sex.’”

### Theme 2: You’re never gonna get these kids back

The second theme, you’re never gonna get these kids back, represents the right to have or not have children. Incarceration had a profound effect on the women’s parental and reproductive rights, including limited contact with their children, barriers to reproduce when and how they wanted, and access to pregnancy and abortion care services.

#### Barriers to abortion

Prior to her incarceration, one woman described how her desire to terminate a pregnancy led her to engage in illegal activity to acquire the necessary money for an abortion. As a result, she was arrested, jailed, and forced to continue the pregnancy until she eventually gave birth while incarcerated:

I wanted to have an abortion. I didn’t want any kids. With that money that they were gonna pay me, I was gonna go have an abortion. I guess God didn’t want me to do it, so I ended up having him. That’s the only reason I did it was the money.

#### Forced abortion

The woman who was coerced into having sex with her court-appointed attorney represents a different example of denied reproductive rights. When the attorney discovered that she was pregnant, he demanded that she have an abortion:

When I stopped answering [his] calls [he stopped representing me]. I had a warrant. I ended up getting caught. I stayed in jail for 6 months. He went to the jail and said, “You wanna try this again, or you wanna go to prison?” Who wants to sit in jail? I got out. Then [the sexual assault] kept on happening. I ended up getting pregnant. Then he made me go get an abortion.

#### Barriers to their children

All the women who did have children described not feeling “fully present” following their arrest: “It affects everybody in such a huge way because you can’t really fully be present. You’re always just so stressed out about what you have to do to care for the kids.” The women further explained that their arrest history and resulting poverty were a constant source of discrimination. Without a social or economic safety net, the women were at risk for involuntary termination of parental rights. One woman described how Child Protective Services had used her history of arrest and being a survivor of sexual abuse as a justification for removing her children from her custody:

[Child Protective Services] would use [my history of surviving sexual abuse] against me. I told them, “Okay, I’ve been having problems. I was sexually abused,” and they would just focus on that. I was drug testing and doing everything right, and they were focused on who was I sleeping with. They treated me really bad and they wouldn’t graduate me. I told them I wanted to take care of my kids, but they shipped them off to [foster care]. I felt like they were just not on my team. They weren’t trying to work with me.

Another woman said that Child Protective Services had terminated her parental rights due to her previous arrest for sex-working, “There was five different women, but they all just said, ‘You’re never gonna get these kids. I’ve never seen anybody with your record get kids from Child Protective Services.’ It felt like they were saying, ‘You’re just not good enough.’” The overwhelming economic stress and discrimination that followed arrest caused the women to live their lives in “survival mode,” which also prevented them from being fully present with their children: “It’s hard because you don’t know what’s gonna happen at any time. When you’re going’ back and forth [to meet the requirements of probation], you can’t work or anything. You’re forced into survival mode.”

#### Barriers to intimate partners

The women also described incarceration-associated discrimination and how it limited their choices of intimate partners, especially when their arrest was due to sex work. This further limited their ability to form healthy relationships, have more children when they wanted to, and receive social support for parenting the children whom they already had. One woman described how difficult it was to find a partner following her arrest:

I feel neglected by society, outcasted, and shunned. Who will take you seriously? Who will date you? Who will wanna marry you? Who will wanna be a father to your child? You have to be all those things, you have to fill all those shoes, but how, and in a timely fashion before your kid’s a teenager?

### Theme 3: why can’t I get out of this hell I’m living in?

The third theme, why can’t I get out of this hell I’m living in? represents the right to parent in safe and sustainable environments. The sexual violence that the women experienced led to the deterioration of their relationships as well as the safety and sustainability of their environments. Moreover, sex work was often forced on to these women after arrest or was a chosen profession out of necessity because the economic barriers following incarceration made survival difficult. These women were forced to do whatever they needed to do to make money and provide for their children at the expense of having to engage in horrible work conditions that further deteriorated their safety, their relationships, and the sustainability of their environment.

#### Lack of a social safety net

They described discrimination because they were single mothers, because they had children outside of marriage, and because they had past histories of arrest and sexual violence. One mother described verbal abuse: “Especially having a child and not being married, [according to my] family, ‘You were sleeping’ around. You only knew this guy for three months before you got pregnant. You’re a whore.” Following arrest, given this absence of a healthy social network, women preferred to self-isolate, which further deteriorated their overall safety. One described suffering from isolation as a mother following arrest:

It’s dark, and it’s painful, and there’s trauma, and you’re so angry. Because I’m remembering all the things that happened that led me there, and I’m thinking, why can’t I just get out of this hell that I’m living in?

Another woman avoided certain people and places. She intentionally wore a ring to indicate that she was married and to avoid being approached by men and further victimized:

I just avoid certain places completely. When I take my daughter places, I go places where there’s only single moms. I try not to wear sexy stuff. I’m not in a relationship, but I wear a wedding ring, so people think I’m married, so guys don’t try to talk to me.

#### Violence in their environment

The women did not want to engage in sex work any longer; however, after encountering the social and economic barriers that followed their arrests, many found themselves with no other choice. Often this work involved men who had perpetuated sexual violence against them in the past. Once again, they found themselves needing to rely on these men for survival. As one woman explained, “I kind of just always had money issues, and I turned to another way to make money. I always fell back into [sex work] every time I would come across hard times, which is why I always ended up incarcerated for the same reason.” Another woman told a similar story: “I turned to [sex work] that I shouldn’t have been doing, and [that] had consequences and repercussions that I had to deal with. It was just a money issue.”

The women felt that the very nature of the criminal justice system forced them to continue with sex work. Several chose “soliciting” because it was a lesser offense than petty theft:

I had stacked up eight felony theft enhancements. Every time I went to jail, my sentences were getting longer and longer. At this point, I’m facing more than 2 years, and I’m just so tired. I’m like, okay. I’m not gonna steal anymore. I’m gonna just go out and solicit myself because I don’t want to go to prison for a very long time. I have actually two solicitation incarcerations—misdemeanors.

One woman described how women charged with “prostitution” are forced to do “whatever they have to do” to support themselves and their children:

A lotta women are incarcerated for prostitution. They have no other option or chance to be able to make money, and yet cannot be around their kids, cannot take care of their kids because they’re judged for that, and can’t get a job. We would work a job if we were able to. We women go through what we have to, to take care of our kids. Whatever we have to.

Another had engaged in sex work for “good” reasons, such as providing for her children despite its being “dangerous.” She stated, “We don’t want to do it. Some will do it for good, like taking care of your kids. It still takes a lot to do it because of the danger. It’s dangerous.” Others echoed those dangers:

It was scary. I just didn’t know who I was gonna run into, or what could possibly happen. ‘Cause anything could possibly happen. It’s just staying out there. I was very scared. I felt alone, real hurt. I became an ugly person, just ‘cause I have a lot of trust issues, really bad trust issues.

This fear was accompanied by a sense of being “helpless,” because there were no other good options for employment:

I felt really ugly with myself. I just never understood why I was going through everything that I was going through. I felt really helpless, hopeless, worthless, angry, hurt. I felt like a really ugly person inside.

Even the women who had never engaged in sex work felt pressured by others to “solicit”:

I did feel pressured [by people in the community] to solicit because the people that I knew would do that. I did feel like, well, I might as well. Never got around to it, and I thank God for it. My thing was the shoplifting. I had to, ‘cause where was I gonna get money from? I didn’t have family helping me.

#### Deterioration of the sustainability of their environment

As a means of coping with the negative emotions and fear that accompanied sex work, many of the women relied on substance use to escape associated trauma: “It was hard doing [sex work] being’ sober ‘cause it’s not normal. I had to be high to do that.” Another woman said,

I started, going to truck stops, and then guys that I knew, like drug dealers, they all wanted to be with me. Then later on in the year, that’s when I started walking the streets. It got to the point that I just wanted money for my next fix. That’s when the heroin kicked in. I needed my heroin to live, to wake up. I would get sick.

Many of the women also felt that sex work increased their need to access healthcare services, yet they avoided healthcare because they feared discrimination. One woman had avoided obtaining essential healthcare because she was too ashamed to reveal her history of sex work to healthcare providers:

Having that solicitation conviction and having exposed myself out there, and just having to relive that sexual abuse that happened to me as a young girl. That really impacted me because, I’ve never been able to really say freely to nobody, and when [doctors and nurses] ask me [about my sexual health], I’m like, “No. No, that’s not true.”

The women also recounted discrimination that they felt to be grounded in misunderstanding yet that still served to silence their voices because they were judged by society:

I think, in society, a lot of people believe that it’s what you asked for. It’s what you deserved. I think it’s just a stigma that sticks there, and people just can’t see past that. It’s so hard because you’re prejudged, and they’re looking at you different.

This lack of understanding, along with the limited dialogue surrounding their lived experiences, kept the women trapped in environments that were often harmful and dangerous. One woman said that society “left her hanging” when she tried to access assistance:

Too often for single moms and people that I know have been arrested, when that time comes when you gather the strength to say something, they just leave you hanging. Society at large does not know how to answer that call because they haven’t heard it. Then they just turn away, and I think that is something that needs to be addressed because there’s got to be a dialogue.

This woman, along with many others in the sample, desired the opportunity to talk about their experiences following incarceration, with the hope of changing the “dialogue” about incarceration at the intersection of mothering. They believed that if people only knew their true circumstances, some of the discrimination and barriers that they had encountered might be resolved.

## Discussion

### Introduction

All twelve of the participants described instances of sexual violence that prevented them full reproductive autonomy following arrest. The findings of this study suggest a critical need for research, practice, and policy to protect the rights of justice-involved women to have complete bodily autonomy, to have or not have children, and to parent their children in safe, sustainable environments.

### Incarceration threatens bodily autonomy

Incarceration pays a role in promoting discrimination against childbearing women of color [7, 12], jeopardizing their right to bodily autonomy [7]. In many ways, our findings are in line with research on this topic. For example, we found that incarceration created insurmountable challenges to women’s social networks, economic stability, access to resources, and the right to fair policies, all of which contribute to incidents of sexual violence that often go underreported and unprosecuted [2, 6, 9]. Similarly, we found that justice-involved women experienced significant discrimination associated with their arrest, substance use, poverty, and sex work, leaving them vulnerable [6, 12]. Further, following incarceration, these women’s right to bodily autonomy was often under attack by those in positions of power and authority. Discrimination, shame, and fear of rearrest left the women exposed to subsequent episodes of sexual violence committed by individuals whom they should have been able to trust.

The application of reproductive justice to the phenomenon of sexual violence and the denial of bodily autonomy demands the evaluation of cultural attitudes, structural barriers, and restrictive policies [2, 7]. Even though the U.S. has recognized sexual violence as a significant barrier to health equity for underrepresented populations [2], associated ongoing inequalities of gender, sexuality, class, and race have served to perpetuate acts of sexual violence [16] like those experienced by the women in our study. O’Neal and Hayes [17] have defined this phenomenon as a “rape culture” supported by patriarchal systems that perpetuate misogyny and the sexual coercion of marginalized individuals while failing to apprehend and stop perpetrators.

Researchers have warned that this “social conditioning” of sexual violence as a norm has historical roots dating back to slavery and to the captivity, control, and exploitation of Black female bodies [3, 9, 16]. Moreover, this historical control and exploitation of women of color is believed by many to be heavily embedded within our modern cultural systems [3, 9, 16]. The findings of our study further support the belief that patriarchal systems protect sexual predators while incriminating, ignoring, or dismissing female survivors of sexual violence [16, 18].

### Incarceration violates fundamental human rights

Incarceration creates social and structural barriers, which contributed to the sexual violence experienced by the women in our study and interfered with their reproductive right to have or not have children. The reproductive justice framework clearly illuminates the violation of these basic, fundamental human rights. The women in our study provided personal examples of such violations, including denial of access to abortion care and coerced pregnancy termination against their will. These findings align well with the literature showing that justice-system involvement violates women’s basic human reproductive rights [14].

The women in our study also described how their carceral experiences prevented them from parenting their children. Economic barriers and punitive policies following arrest and/or probation caused “constant worry,” which prevented them from being “fully present” in their children’s lives. Other researchers have shown that the intersection of incarceration and motherhood jeopardizes the way in which women live, interact with others, and parent their children [16]. For example, in comparison with justice-involved men, justice-involved women are more likely to be single, primary caregivers of young children, often relying on next of kin to care for their children during incarceration. This places their children at greater risk for entering the foster care system [16].

In the U.S., deeply engrained gendered norms, biases, and expectations of good mothers are often framed within a white, middle-class narrative that fails to recognize other social constructs [16, 18]. These preconceived ideas about good mothers were problematic for the women in our study, who faced a child welfare system that rarely considered the childrearing values, beliefs, or rights of the women as parents yet had substantial power over decisions to terminate parental rights. Previous research also indicates that termination of parental rights is high among justice-involved women even before incarceration. This may be due to an overlap or cross-reporting between child welfare and the criminal justice system that can result in repeated investigations over a span of months to years [19]. Thus, social and economic vulnerabilities caused by incarceration [12, 19] serve to further restrict women’s opportunities to parent their children, owing to their failure to conform with society’s views of traditional women or good mothers [16, 19].

A unique finding of our study is the social isolation described by the women who were involved in sex work. Most of the women did engage in this work out of financial necessity and a need to provide for their children, but the consequences were substantial, jeopardizing their reproductive rights by limiting their access to potentially stable partners. This isolation also limited their opportunities to build social support networks to assist with parenting. Moreover, the discrimination and violence often associated with sex work further limited their ability to rebuild past supportive relationships as they self-isolated for protection.

Other researchers have found sex work to be socially isolating [18, 20] but they have not studied it in populations influenced by incarceration. From a societal perspective, women who are sex workers and control their sexuality for financial stability deviate from society’s preconceived notions of traditional women [18, 20]. Women who engage in sex work fail to conform to traditional gender norms of womanhood, securing financial stability without marriage [18]. Such women have been shunned by society for taking control of their sexuality, whether they do so for economic stability (i.e., sex work) or for their own pleasure, because women’s bodies have been viewed historically for the purpose of men’s pleasure or for reproduction [18, 20].

### Cruel and unusual punishment of women following arrest

According to the Eighth Amendment of the U.S. constitution, “cruel and unusual punishment” with “excessive fines and bail” is unlawful [21]. Nevertheless, the women in our study, like previous researchers [14, 22], described unreasonable requirements and unaffordable fines that make parenting in a safe and sustainable environment nearly impossible after incarceration. The women’s meager incomes were often rapidly depleted by the expenses of their probation, such as weekly urinalyses, supervision fees, transportation, and childcare. The income of most of our participants was less than or equal to $9000 per year, similar to the $10,000 national average for all justice-involved women. Further, national statistics indicate that incarceration-associated penalties and fines are equivalent to $10,000 [8, 22], roughly an entire year’s income for these women or greater. This financial burden often results in extreme poverty and/or homelessness for justice-involved women, and it leads one to question whether such hardship might be unconstitutional [21].

Failure to comply with the monetary requirements of probation often leads to rearrest, and it can contribute to the termination of parental rights. The women in our study did whatever was necessary to meet these financial demands while parenting their children, including sex work. This phenomenon has been previously reported in literature that cites the interwoven nature of the sex industry and motherhood in underrepresented populations, where few sources of income are available to parent in safe, sustainable environments [23]. The safety of the women in our study was further compromised by the violent nature of sex work, and many turned to substances to help them cope with this danger. This finding has been previously reported [17], and the consequences of such coping mechanisms often resulted in further separation from children and the unraveling of the safety and substantiality of parenting environments.

The findings of our study also reflect a general lack of effort to address the systems that perpetuate sexual violence in the lives of our nation’s most vulnerable women, such as the criminal justice system. For example, research shows that women of color with less economic stability and power, such as those who have been arrested or involved in the sex work industry, experience a disproportionate rate of sexual violence [2, 6]. Yet experts suggest that this group of women seems to have been forgotten in the U.S. rape narrative, which focuses predominately on the affluent white [6]. The participants in our study corroborated this suspicion through their stories of coercion, sexual violence, and silencing. Despite laws such as the 1994 Violence Against Women Act, President Obama’s Executive order in 2012 related to gender-based violence, and the more recent “Me too” movement, the prosecution and prevention of such crimes in underrepresented groups has lagged [24, 25]. The findings of our study align with previous research showing that the criminalization of sex work contributes to numerous reproductive and human rights violations [23]. The decriminalization of sex work might decrease sexual, intimate partner, and workplace violence, as well as the infringement of human and reproductive rights that further compromise safe, sustainable environments for parenting particularly in underrepresented populations [23].

### Public health implications

The World Health Organization has suggested that universal healthcare could play a crucial role in eliminating violence against women [1]. Gender-specific and trauma-informed healthcare delivery models could provide women who are survivors of sexual violence with access to preventative and life-saving care [1, 26]. Meaning, models that recognize the pathways that lead women to become incarcerated and use substances is usually from experiencing violence [1, 26]. Further, care models that keep women and children together, and aid in harm reduction such as realizing substance use is a disease, not a crime, and applying de-stigmatizing language within their care models [1, 26]. Many of the women who participated in our study described how sexual violence had jeopardized their health. Even when healthcare was available through emergency departments or the county jail, shame and discrimination associated with sex work and sexual violence prevented the women from accessing services that they needed.

To address these issues, more recognition about how incarceration is a driver of sexual violence against women is needed with more attention in harm reduction and addressing the environments in which women live is required. The World Health Organization recommends the RESPECT framework to guide healthcare models in addressing sexual violence against women [2]. RESPECT stands for relationship skills strengthening; empowerment of women; services ensured; poverty reduction; enabling environments (schools, workplaces, public spaces) created; child and adolescent abuse prevention; and transformed attitudes, beliefs, and norms [1].

This model incorporates social and economic stability through (a) education related to sexual violence; (b) investment in educational systems, particularly for women and girls; (c) safe, sustainable living environments through mandates protecting vulnerable groups and alleviating redlining; (d) equal pay and cash transfers for underserved women; (e) gender-specific programs and care models specific to the needs of women and mothers; and (f) a transformation in the normative attitudes surrounding sexuality and gender by partnering with survivors and stakeholders on community, societal, national, and global levels to reform policy, attitudes, and research methods [1].

This aforementioned framework calls for more precise measures to quantify sexual violence that can be translated to inform greater cultural awareness [2]. Data sharing across systems (probation, jail, healthcare, substance use treatment, and Child Protective Services), counties, and states is needed to bring sexual predators to justice. Employees of organizations and systems involved in the criminal justice system such as county jails, county hospitals, probation, pretrial bond, medication assistance therapy, and Child Protective Services must have gender-specific, trauma-informed training. Regular client assessments should include an appraisal of sexual violence experiences in a safe, judgement-free environment.

Finally, individuals belonging to underrepresented populations must have a seat at the table during conversations about how best to end the cycle of sexual violence. Our study has focused on a single group, Latina mothers influenced by incarceration; but more research is needed to better understand the experiences of sexual violence in other groups such as the lesbian, gay, bisexual, transgender, queer or questioning, intersex, pansexual, two-spirit, androgenous, or asexual justice-involved communities [27]. Investigations into same-sex perpetrators are also essential.

### Summary

This was a secondary analysis that used the reproductive justice framework to illuminate the experiences of Latina mothers following arrest. This study highlights how incarceration whether being detained in jail or mandated to be on community supervision which includes parole, probation, or medication assistance therapy may act as a driver for sexual violence in underserved women which puts limits on their bodily autonomy, right to have children when or how they want, and be able to parent the children they choose to have in environments that are sustainable and free from violence.

### Limitations

One inherent limitation was the use of the telephone. Although all of the women consented they were in a private and secluded area during the interview process, there are risks that the participants reframed from disclosing information in fear they would be overheard by someone in close proximity. However, the interviews lasted 60–120 minutes which led to data saturation and suggested the women seemed comfortable disclosing very personal and detailed information about sexual violence in their own private spaces. We also found the use of the telephone alleviated participant burden and was convenient for the women to balance the responsibilities of motherhood and tasks mandated by their community supervision.

Another limitation was that recruitment was limited to one geographical location in Texas because of the COVID-19 pandemic, however, we sampled from multiple sites which diversified the data we were able to retrieve. Moreover, there is always a possibility of social desirability or recall bias, however, the use of the telephone gave an extra component of confidentiality that made the participants feel more comfortable being in their own environment without being face to face with the interviewer to disclose their past. In addition, we used probe questions and follow-up interviews in instances that needed more explanation from the participants.

## Conclusion

In this secondary data analysis of sexual violence experienced by Latina mothers impacted by incarceration, the reproductive justice framework has enabled us to focus on women’s right to bodily autonomy, their right to have or not have children, and their right to parent their children in safe and sustainable environments. Our findings reveal the negative influence of incarceration on women’s reproductive and civil rights. This research is a step toward addressing gaps in the literature on sexual violence at the intersection of incarceration and underrepresented childbearing women. More research is needed to understand the social, economic, and political barriers that perpetuate sexual violence. Our findings suggest that universal healthcare, participatory research, changing cultural mindsets, decriminalization of sex work, and more comprehensive tracking and prosecution of sexual predators may be key to ending sexual violence in justice-involved women.

## Data Availability

All data generated or analyzed during this study are included in this published dissertation [and its supplementary information files]: Crawford, A. D. (2021). *The Experiences of Latina Mothers Impacted by Incarceration* (Order No. 28542845). ProQuest Dissertations & Theses Global. https://www.proquest.com/docview/2564587357?pq-origsite=gscholar&fromopenview=true
